# A Rare Case of Primary Anorectal Malignant Melanoma

**DOI:** 10.7759/cureus.15474

**Published:** 2021-06-06

**Authors:** Sandeep Bhattarai, Oseen Shaikh, Naveen Kumar Gaur, Muhamed Tajudeen, Uday Kumbhar

**Affiliations:** 1 Surgery, Jawaharlal Institute of Postgraduate Medical Education and Research, Puducherry, IND

**Keywords:** melanoma, anal canal, rectum, malignant, abdominoperineal resection

## Abstract

Malignant melanoma affecting the anorectum is very rare. We present a 63-year-old female who presented with features of bleeding per rectum and painful defecation. On examination, the patient had a palpable mass on the right side of the anorectum, with predominant exophytic growth and intraluminal extension. Biopsy and imaging studies were diagnostic of malignant melanoma. The patient was discussed on the tumor board and planned for abdominoperineal resection. Postoperatively, the patient was started on chemotherapy. The patient was followed up for two years, and there was no evidence of any recurrence.

## Introduction

Malignant melanoma affecting the gastrointestinal tract is very rare, and the anorectum is the most common site being affected. Anorectal malignant melanoma (ARMM) accounts for less than one percent of colorectal malignancies [1]. The exact etiology of the disease is not known. Patients usually present with non-specific symptoms like painful defecation and bleeding per rectum, and hence early diagnosis is usually tricky. Many of the time, it is misdiagnosed as hemorrhoid or polyp. Computed tomography (CT) and magnetic resonance imaging (MRI) helps in the diagnosis of the ARMM. Histopathology and immunohistochemistry are the definitive modes of the diagnosis of the ARMM. Treatments of the ARMM include surgery like abdominoperineal resection (APR) and wide local excision (WLE), immunotherapy, and chemotherapy [2]. The prognosis of the ARMM is usually poor. We present a 63-year female diagnosed with ARMM, and she was managed by APR and adjuvant chemotherapy.

## Case presentation

A 63-year-old female presented to us with complaints of bleeding per rectum from the last one month along with painful defecation since last five days. There was a history of loss of weight and appetite but was not able to quantify. General physical examination was unremarkable and abdominal examination was normal. Per rectal examination showed 5 cm x 4 cm firm mass, which was 2 cm from the anal verge, predominantly on the right side. The upper limit of the mass was not felt, and the mass was not infiltrating the vagina.

**Figure 1:**
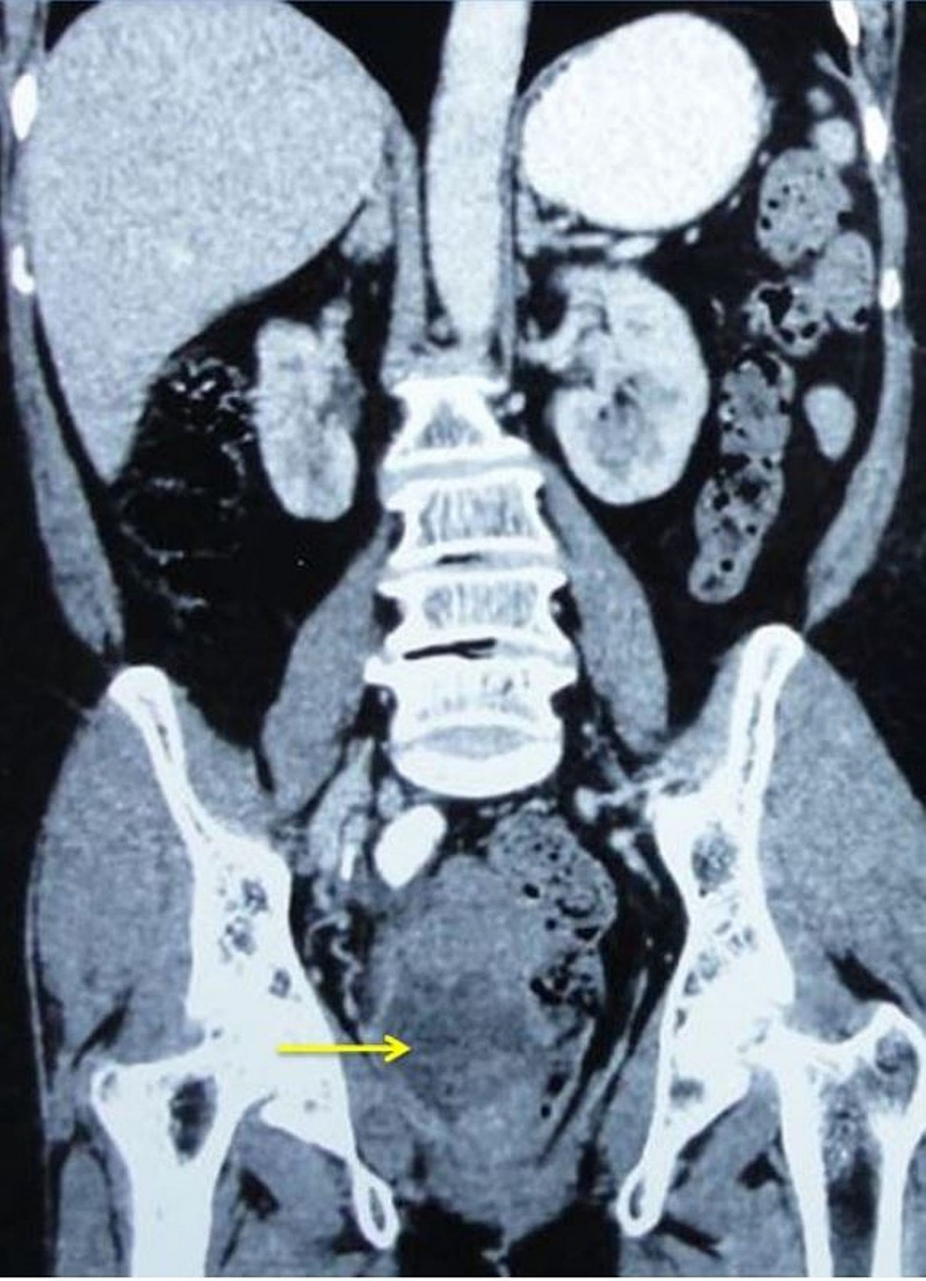
Computed tomography showing growth in the rectal wall (arrow), predominantly exophytic, causing thinning of the mesorectum, without any evidence of intra-abdominal metastasis.

Routine blood investigations, including hemoglobin, renal function test (RFT), and liver function test (LFT), were normal. Carcinoembryonic antigen (CEA) was 1.2 ng/mL. Ultrasound abdomen did not show any evidence of liver metastasis or any lymphadenopathy. CT abdomen and pelvis showed a large lobulated infiltrative mass lesion in the rectum measuring 9.7 cm x 8 cm x 7 cm arising from right lateral, right anterior, and right posterior wall, starting at 2.8 cm from the anal verge. The mass appears to be heterogeneous with areas of necrosis. The large exophytic component is causing thinning of the right mesorectum (Figure 1).

**Figure 2:**
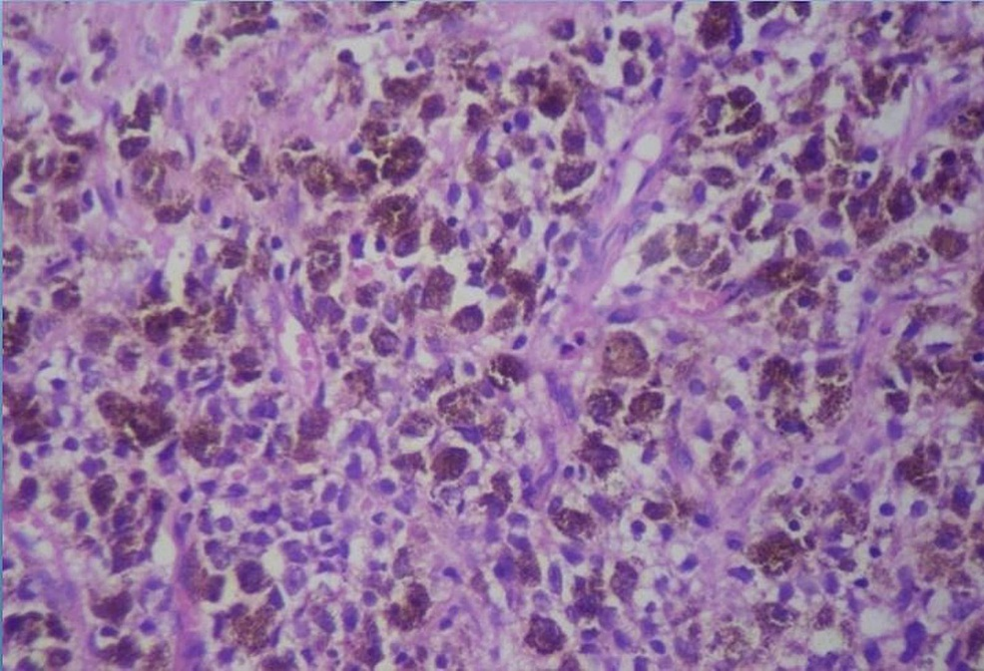
Histopathology image showing the presence of round to polygonal cells with melanin pigment.

There was no evidence of any metastasis in the abdomen or inguinal lymph nodes. MRI abdomen and pelvis showed a large mass lesion involving the rectal wall and anorectal junction with intraluminal component (4.6 cm x 3 cm) and exophytic component (8.5 cm x 5 cm). This mass lesion was hyperintense on the T1 phase, suggestive of rectal melanoma, without any evidence of metastasis anywhere. Proctoscopic biopsy of the mass was done, which showed the presence of round to polygonal cells with melanin pigment (Figure 2).

On immunohistochemistry, they were found to be positive for human melanoma black-45 (HMB-45) and S100 staining.

**Figure 3:**
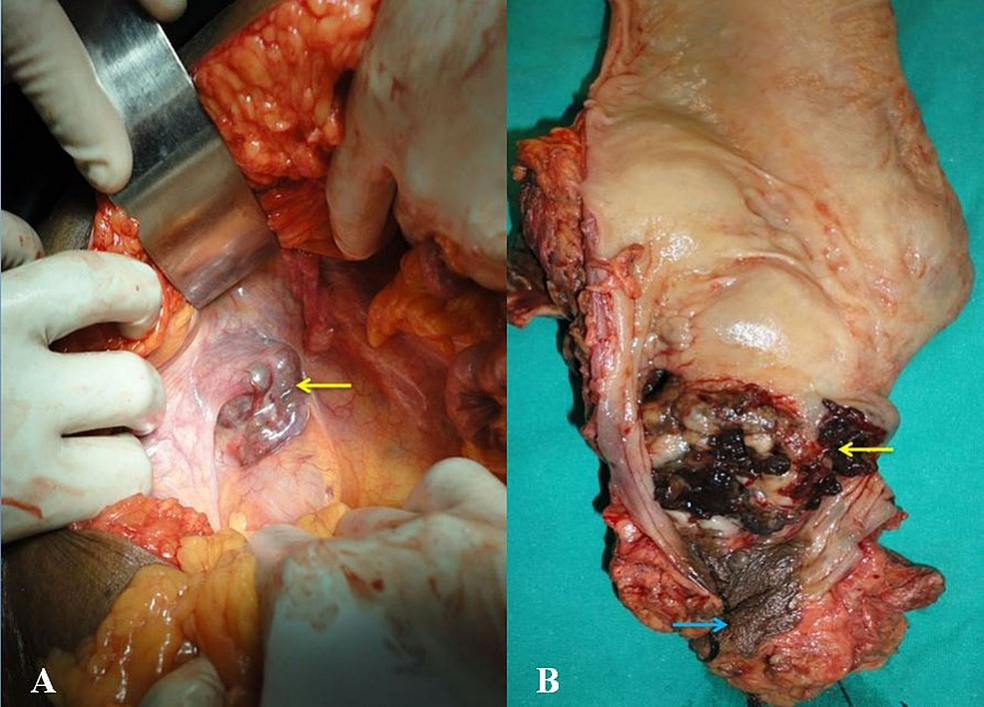
Intraoperative image showing (A) growth in the rectum (arrow) with blackish hue and (B) abdominoperineal resection specimen showing blackish growth at the anorectal junction (yellow arrow) and distal anal canal (blue arrow).

The patient was discussed on the tumor board and planned for surgical management. The patient underwent APR and permanent colostomy (Figures 3A, 3B).

Postoperatively, the patient recovered without any complications. The patient was started on a carboplatin and paclitaxel-based chemotherapy regimen. The patient was followed up for the next two years, and there was no evidence of any recurrence.

## Discussion

ARMM is a highly malignant tumor of the rectum, and usually, the prognosis of the disease is not good. The anal canal is the most common site of malignant melanoma in the gastrointestinal tract because melanocytes are commonly found in the anal squamous epithelium. ARMM accounts for less than one percent of rectal tumors [1]. These tumors are more common in females than males and are usually seen after 60 years. ARMM is usually located within 6 cm of the anal rim [3]. Our patient was a female diagnosed with ARMM in the sixth decade of life.

The exact pathogenesis of the disease is not known. However, are several theories proposed; none has been fully validated. Few have suggested that these originate from the neural crest cells, and few have suggested that they arise from the intestinal Schwann cells. Although murine sarcoma viral oncogene homolog B1 (BRAF) gene mutations are common in cutaneous melanoma, these are uncommon in ARMM. There is a higher incidence of the proto-oncogene tyrosine-protein kinase kit (KIT) oncogene mutation in ARMM.

Patients with ARMM may have varied clinical features and presentations. The patient may present with bleeding per rectum, proctalgia, pruritis, and changes in bowel habits. In a few, it presents as hemorrhoids and skin tags, and in these patients, diagnosis of the ARMM may be incidental after the excision of hemorrhoid or the skin tag. Such scenarios happen in almost about 20% of the patients with ARMM [4,5]. Many patients present with inguinal lymph nodes or liver metastasis. On examination, patients may have palpable growth with typical pigmentation. Few may have a polyp or hemorrhoid-like presentation. For patients with amelanocytic melanoma, diagnosis is challenging, as these patients are usually initially misdiagnosed as basal cell carcinoma, adenocarcinoma, pyogenic granuloma, or Bowen’s disease [6]. Our patient had presented with bleeding per rectum and painful defecation. The growth was predominantly exophytic with the intraluminal component.

Diagnosis of ARMM is made by biopsy and is considered the gold standard method of diagnosing ARMM. Proctoscopy or colonoscopic biopsies are usually taken. A colonoscopy helps characterize the lesion, including the site, surface, color, and invasion of the dentate line. Histopathological examination is the gold standard for the diagnosis of ARMM. The cells are usually epitheloid or pleomorphic [7]. These tumors are usually positive for S100, HMB-45, and melanin A immunohistochemical marker. Our patient had undergone a biopsy; histopathology and immunohistochemistry were consistent with the diagnosis of ARMM.

Imaging studies help to stage the disease and guide the management of the patients diagnosed with ARMM. Ultrasound abdomen may show the presence of liver metastasis in patients with advanced ARMM. CT abdomen and pelvis are usually done to assess the extent of the disease in patients suspected to have neoplasia. ARMM is usually seen as bulky intraluminal fungating masses, focally expanding, obscuring the lumen, without causing an obstruction. There is usually the presence of the perirectal fat stranding and enlarged lymph nodes [8]. MRI abdomen and pelvis are also very helpful for staging the disease and planning for the treatment. The finding of MRI usually correlated with histopathology. ARMM is usually hyperintense on T1-weighted images and hypointense on T2-weighted images. This is due to the paramagnetic property of the melanotic component, which shortens the T1 relaxation time and increases the T2 relaxation time. However, these features may be absent in patients who have amelanotic melanoma [9]. Positron emission tomography-CT (PET-CT) helps stage for the disease and to assess the response of the disease to the therapy. It also helps in detecting distant metastasis from malignant melanoma [2]. Our patent had undergone an MRI abdomen and pelvis, which was consistent with a diagnosis of ARMM.

Although there are no specific markers for ARMM, several serum markers are used in patients with ARMM. These include lactate dehydrogenase (LDH), melanoma-inhibiting activity (MIA), enolase, and chitinase 3-like-1 (YKL-40) [2]. ARMM is usually radioresistant and radiotherapy does not have a significant role in the management. Treatment of the ARMM depends on the stage of the disease and it includes surgery, targeted therapy, and immunotherapy [2]. Surgical treatment of the ARMM includes APR, WLE, endoscopic mucosal resection (EMR), depending on the stage of the disease. Palliative surgery is done in patients if the tumor is not resectable and has metastasis. Multiple chemotherapy regimens are used in patients with ARMM. The common chemotherapy drugs used are dacarbazine and a combination of carboplatin and paclitaxel. Immunotherapy, like the use of interferon-alpha and interleukin-2, has been used in patients with metastatic disease. Tyrosine kinase (TK) inhibitors (imatinib) and anti-cytotoxic T-lymphocyte-associated antigen 4 (CTLA4) inhibitors (ipilimumab) have also been used. The prognosis of the patients with ARMM is poor, and five-year survival is hardly found to be 20% [2]. This is probably because of delay in diagnosis, inherent aggressiveness of the disease, and earlier spread along the lymphatics. Our patient was treated with APR and adjuvant chemotherapy.

## Conclusions

Malignant melanoma of the anorectum is very rare. Patients present with non-specific clinical features hence, early diagnosis is usually difficult. Definitive diagnosis is done by histopathology and immunohistochemistry. Patients diagnosed with ARMM are targeted with surgery. Other modalities of the treatment are available but helpful in metastatic disease. The prognosis of the patient diagnosed with ARMM is usually poor.
